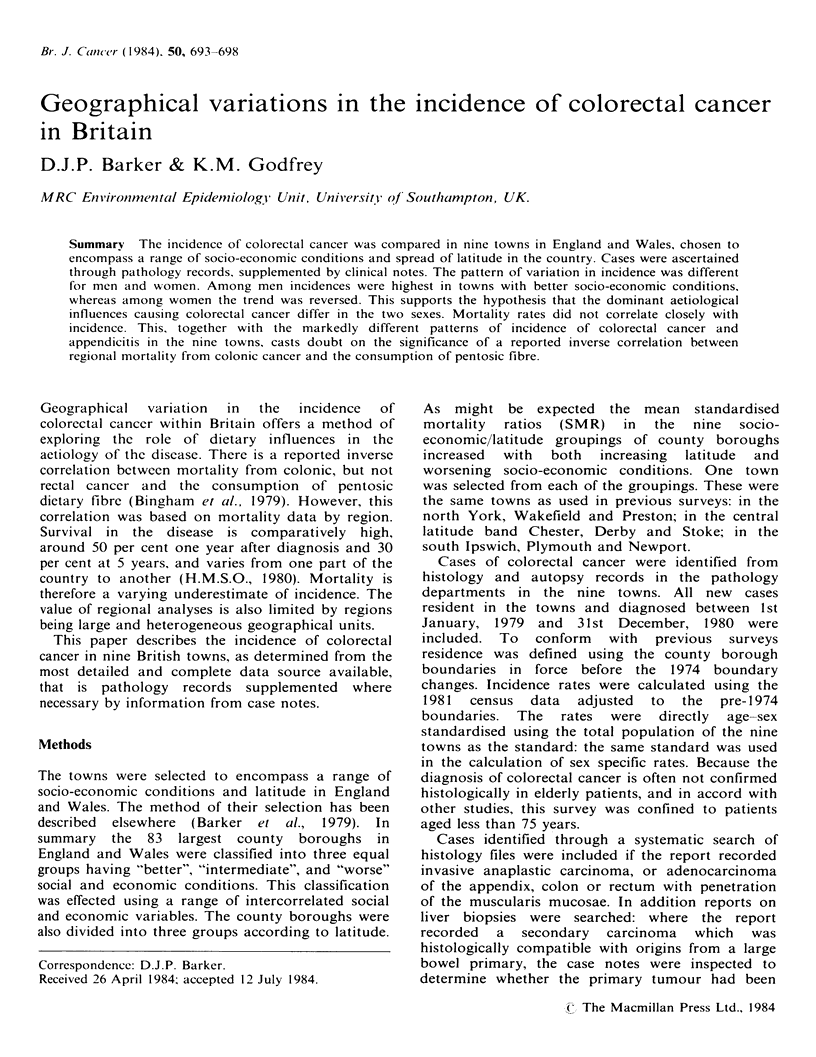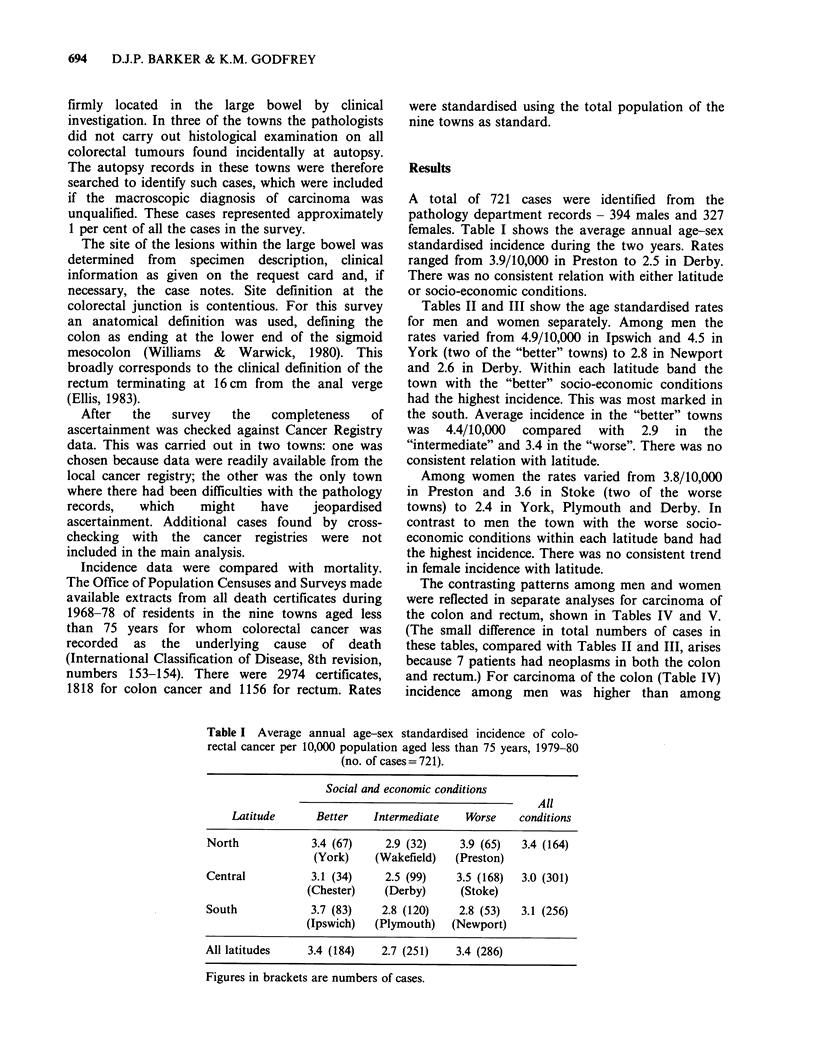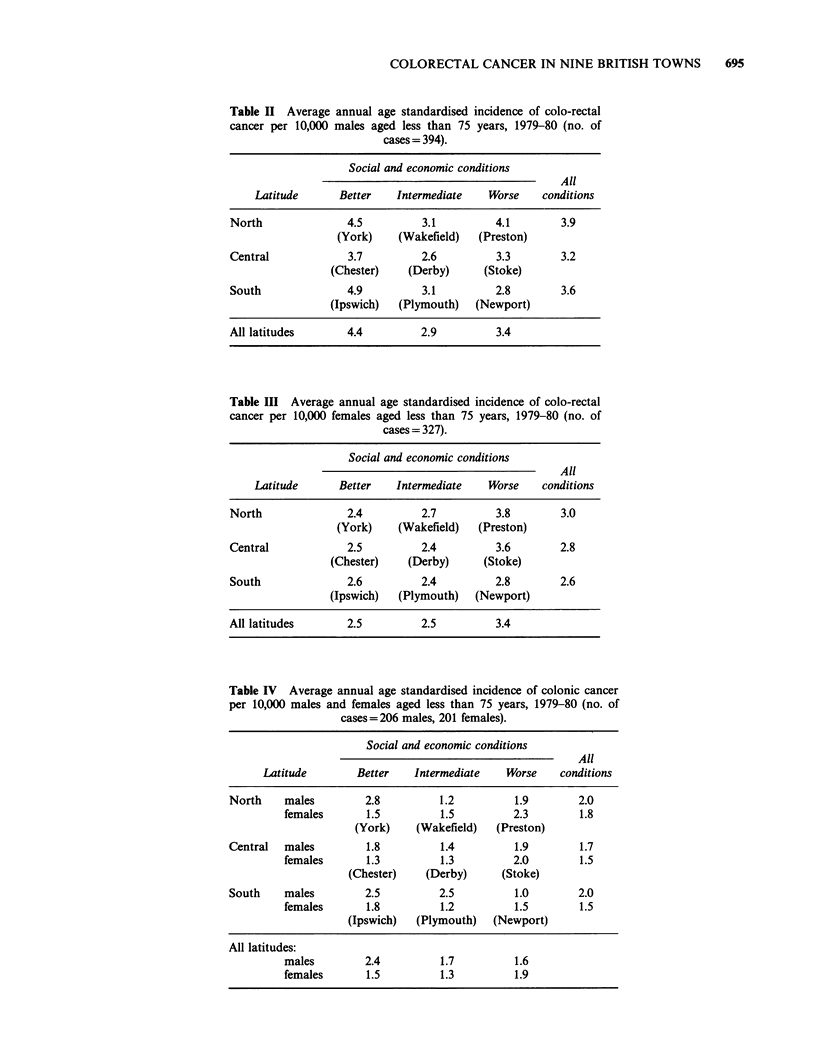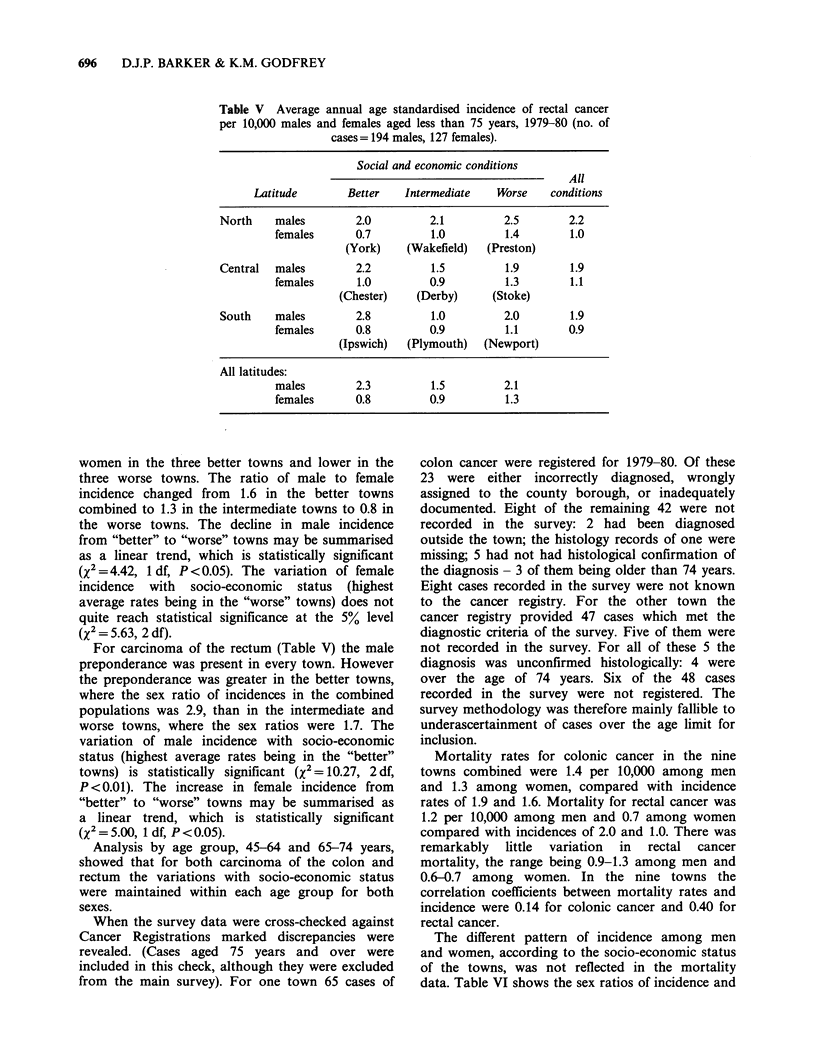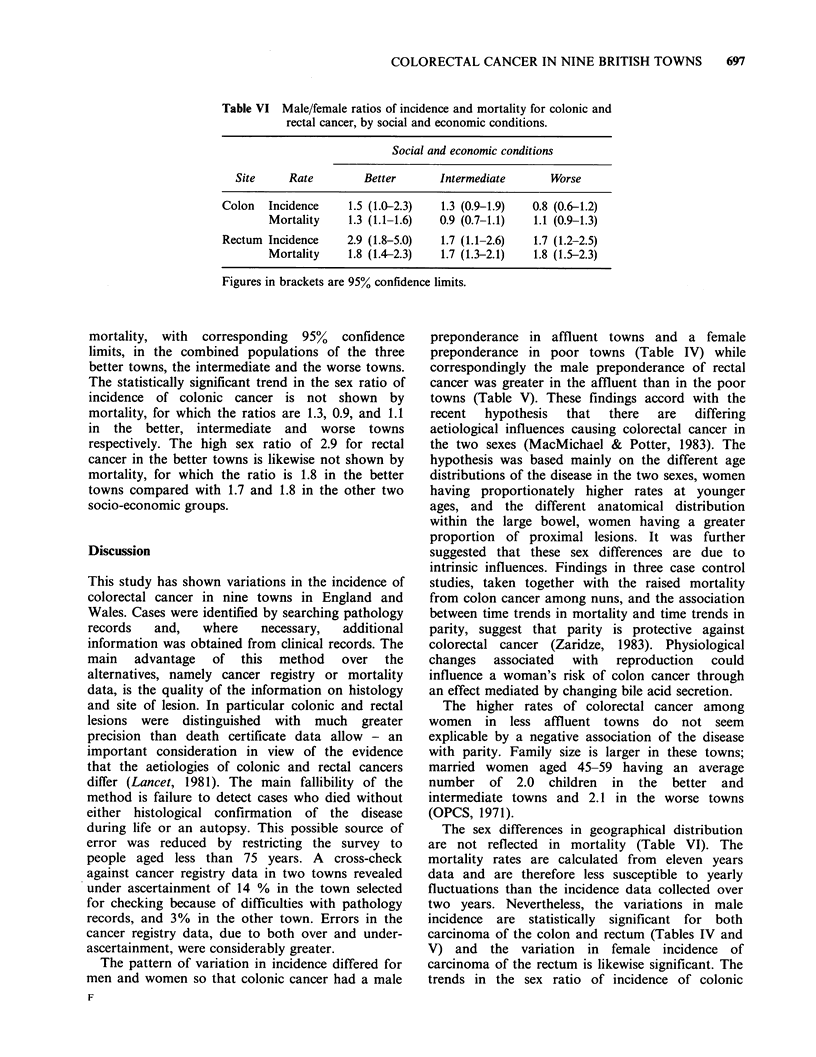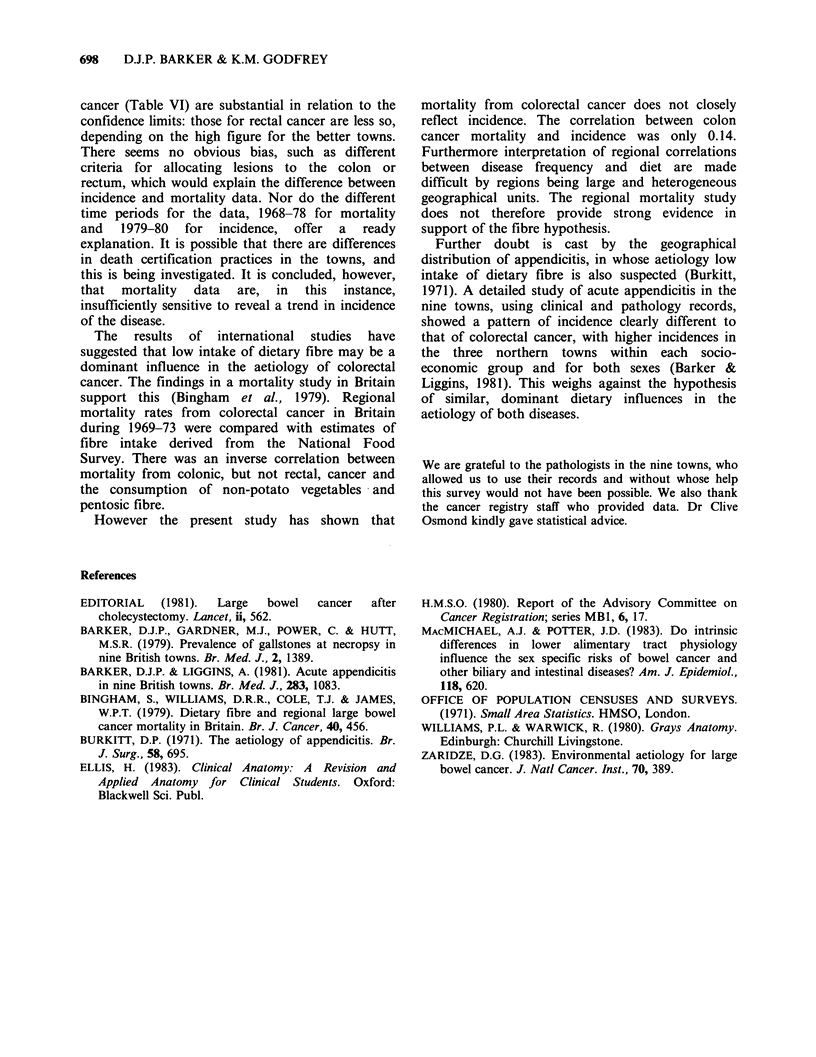# Geographical variations in the incidence of colorectal cancer in Britain.

**DOI:** 10.1038/bjc.1984.238

**Published:** 1984-11

**Authors:** D. J. Barker, K. M. Godfrey

## Abstract

The incidence of colorectal cancer was compared in nine towns in England and Wales, chosen to encompass a range of socio-economic conditions and spread of latitude in the country. Cases were ascertained through pathology records, supplemented by clinical notes. The pattern of variation in incidence was different for men and women. Among men incidences were highest in towns with better socio-economic conditions, whereas among women the trend was reversed. This supports the hypothesis that the dominant aetiological influences causing colorectal cancer differ in the two sexes. Mortality rates did not correlate closely with incidence. This, together with the markedly different patterns of incidence of colorectal cancer and appendicitis in the nine towns, casts doubt on the significance of a reported inverse correlation between regional mortality from colonic cancer and the consumption of pentosic fibre.


*Br. J. Cancer* (1984), **50**, 693–698

### Geographical variations in the incidence of colorectal cancer in Britain

D.J.P. Barker & K.M. Godfrey

*MRC Environmental Epidemiology Unit, University of Southampton, UK.*

**Summary** The incidence of colorectal cancer was compared in nine towns in England and Wales, chosen to encompass a range of socio-economic conditions and spread of latitude in the country. Cases were ascertained through pathology records, supplemented by clinical notes. The pattern of variation in incidence was different for men and women. Among men incidences were highest in towns with better socio-economic conditions, whereas among women the trend was reversed. This supports the hypothesis that the dominant aetiological influences causing colorectal cancer differ in the two sexes. Mortality rates did not correlate closely with incidence. This, together with the markedly different patterns of incidence of colorectal cancer and appendicitis in the nine towns, casts doubt on the significance of a reported inverse correlation between regional mortality from colonic cancer and the consumption of pentosic fibre.

Geographical variation in the incidence of colorectal cancer within Britain offers a method of exploring the role of dietary influences in the aetiology of the disease. There is a reported inverse correlation between mortality from colonic, but not rectal cancer and the consumption of pentosic dietary fibre (Bingham *et al.*, 1979). However, this correlation was based on mortality data by region. Survival in the disease is comparatively high, around 50 per cent one year after diagnosis and 30 per cent at 5 years, and varies from one part of the country to another (H.M.S.O., 1980). Mortality is therefore a varying underestimate of incidence. The value of regional analyses is also limited by regions being large and heterogeneous geographical units.

This paper describes the incidence of colorectal cancer in nine British towns, as determined from the most detailed and complete data source available, that is pathology records supplemented where necessary by information from case notes.

### Methods

The towns were selected to encompass a range of socio-economic conditions and latitude in England and Wales. The method of their selection has been described elsewhere (Barker *et al.*, 1979). In summary the 83 largest county boroughs in England and Wales were classified into three equal groups having "better", "intermediate", and "worse" social and economic conditions. This classification was effected using a range of intercorrelated social and economic variables. The county boroughs were also divided into three groups according to latitude.

Correspondence: D.J.P. Barker.
Received 26 April 1984; accepted 12 July 1984.

As might be expected the mean standardised mortality ratios (SMR) in the nine socio-economic/latitude groupings of county boroughs increased with both increasing latitude and worsening socio-economic conditions. One town was selected from each of the groupings. These were the same towns as used in previous surveys: in the north York, Wakefield and Preston; in the central latitude band Chester, Derby and Stoke; in the south Ipswich, Plymouth and Newport.

Cases of colorectal cancer were identified from histology and autopsy records in the pathology departments in the nine towns. All new cases resident in the towns and diagnosed between 1st January, 1979 and 31st December, 1980 were included. To conform with previous surveys residence was defined using the county borough boundaries in force before the 1974 boundary changes. Incidence rates were calculated using the 1981 census data adjusted to the pre-1974 boundaries. The rates were directly age–sex standardised using the total population of the nine towns as the standard: the same standard was used in the calculation of sex specific rates. Because the diagnosis of colorectal cancer is often not confirmed histologically in elderly patients, and in accord with other studies, this survey was confined to patients aged less than 75 years.

Cases identified through a systematic search of histology files were included if the report recorded invasive anaplastic carcinoma, or adenocarcinoma of the appendix, colon or rectum with penetration of the muscularis mucosae. In addition reports on liver biopsies were searched: where the report recorded a secondary carcinoma which was histologically compatible with origins from a large bowel primary, the case notes were inspected to determine whether the primary tumour had been

© The Macmillan Press Ltd., 1984

firmly located in the large bowel by clinical investigation. In three of the towns the pathologists did not carry out histological examination on all colorectal tumours found incidentally at autopsy. The autopsy records in these towns were therefore searched to identify such cases, which were included if the macroscopic diagnosis of carcinoma was unqualified. These cases represented approximately 1 per cent of all the cases in the survey.

The site of the lesions within the large bowel was determined from specimen description, clinical information as given on the request card and, if necessary, the case notes. Site definition at the colorectal junction is contentious. For this survey an anatomical definition was used, defining the colon as ending at the lower end of the sigmoid mesocolon (Williams & Warwick, 1980). This broadly corresponds to the clinical definition of the rectum terminating at 16 cm from the anal verge (Ellis, 1983).

After the survey the completeness of ascertainment was checked against Cancer Registry data. This was carried out in two towns: one was chosen because data were readily available from the local cancer registry; the other was the only town where there had been difficulties with the pathology records, which might have jeopardised ascertainment. Additional cases found by cross-checking with the cancer registries were not included in the main analysis.

Incidence data were compared with mortality. The Office of Population Censuses and Surveys made available extracts from all death certificates during 1968–78 of residents in the nine towns aged less than 75 years for whom colorectal cancer was recorded as the underlying cause of death (International Classification of Disease, 8th revision, numbers 153–154). There were 2974 certificates, 1818 for colon cancer and 1156 for rectum. Rates were standardised using the total population of the nine towns as standard.

### Results

A total of 721 cases were identified from the pathology department records – 394 males and 327 females. Table I shows the average annual age–sex standardised incidence during the two years. Rates ranged from 3.9/10,000 in Preston to 2.5 in Derby. There was no consistent relation with either latitude or socio-economic conditions.

Tables II and III show the age standardised rates for men and women separately. Among men the rates varied from 4.9/10,000 in Ipswich and 4.5 in York (two of the "better" towns) to 2.8 in Newport and 2.6 in Derby. Within each latitude band the town with the "better" socio-economic conditions had the highest incidence. This was most marked in the south. Average incidence in the "better" towns was 4.4/10,000 compared with 2.9 in the "intermediate" and 3.4 in the "worse". There was no consistent relation with latitude.

Among women the rates varied from 3.8/10,000 in Preston and 3.6 in Stoke (two of the worse towns) to 2.4 in York, Plymouth and Derby. In contrast to men the town with the worse socio-economic conditions within each latitude band had the highest incidence. There was no consistent trend in female incidence with latitude.

The contrasting patterns among men and women were reflected in separate analyses for carcinoma of the colon and rectum, shown in Tables IV and V. (The small difference in total numbers of cases in these tables, compared with Tables II and III, arises because 7 patients had neoplasms in both the colon and rectum.) For carcinoma of the colon (Table IV) incidence among men was higher than among

**Table I** Average annual age–sex standardised incidence of colorectal cancer per 10,000 population aged less than 75 years, 1979–80 (no. of cases = 721).

| | *Social and economic conditions* | | | |
|---|---|---|---|---|
| *Latitude* | *Better* | *Intermediate* | *Worse* | *All conditions* |
| North | 3.4 (67) (York) | 2.9 (32) (Wakefield) | 3.9 (65) (Preston) | 3.4 (164) |
| Central | 3.1 (34) (Chester) | 2.5 (99) (Derby) | 3.5 (168) (Stoke) | 3.0 (301) |
| South | 3.7 (83) (Ipswich) | 2.8 (120) (Plymouth) | 2.8 (53) (Newport) | 3.1 (256) |
| All latitudes | 3.4 (184) | 2.7 (251) | 3.4 (286) | |

Figures in brackets are numbers of cases.

**Table II** Average annual age standardised incidence of colo-rectal cancer per 10,000 males aged less than 75 years, 1979–80 (no. of cases = 394).

| | *Social and economic conditions* | | | |
|---|---|---|---|---|
| *Latitude* | *Better* | *Intermediate* | *Worse* | *All conditions* |
| North | 4.5 (York) | 3.1 (Wakefield) | 4.1 (Preston) | 3.9 |
| Central | 3.7 (Chester) | 2.6 (Derby) | 3.3 (Stoke) | 3.2 |
| South | 4.9 (Ipswich) | 3.1 (Plymouth) | 2.8 (Newport) | 3.6 |
| All latitudes | 4.4 | 2.9 | 3.4 | |

**Table III** Average annual age standardised incidence of colo-rectal cancer per 10,000 females aged less than 75 years, 1979–80 (no. of cases = 327).

| | *Social and economic conditions* | | | |
|---|---|---|---|---|
| *Latitude* | *Better* | *Intermediate* | *Worse* | *All conditions* |
| North | 2.4 (York) | 2.7 (Wakefield) | 3.8 (Preston) | 3.0 |
| Central | 2.5 (Chester) | 2.4 (Derby) | 3.6 (Stoke) | 2.8 |
| South | 2.6 (Ipswich) | 2.4 (Plymouth) | 2.8 (Newport) | 2.6 |
| All latitudes | 2.5 | 2.5 | 3.4 | |

**Table IV** Average annual age standardised incidence of colonic cancer per 10,000 males and females aged less than 75 years, 1979–80 (no. of cases = 206 males, 201 females).

| | | *Social and economic conditions* | | | |
|---|---|---|---|---|---|
| *Latitude* | | *Better* | *Intermediate* | *Worse* | *All conditions* |
| North | males | 2.8 | 1.2 | 1.9 | 2.0 |
| | females | 1.5 | 1.5 | 2.3 | 1.8 |
| | | (York) | (Wakefield) | (Preston) | |
| Central | males | 1.8 | 1.4 | 1.9 | 1.7 |
| | females | 1.3 | 1.3 | 2.0 | 1.5 |
| | | (Chester) | (Derby) | (Stoke) | |
| South | males | 2.5 | 2.5 | 1.0 | 2.0 |
| | females | 1.8 | 1.2 | 1.5 | 1.5 |
| | | (Ipswich) | (Plymouth) | (Newport) | |
| All latitudes: | | | | | |
| | males | 2.4 | 1.7 | 1.6 | |
| | females | 1.5 | 1.3 | 1.9 | |

**Table V** Average annual age standardised incidence of rectal cancer per 10,000 males and females aged less than 75 years, 1979–80 (no. of cases = 194 males, 127 females).

| *Latitude* | | *Social and economic conditions* *Better* | *Intermediate* | *Worse* | *All conditions* |
|---|---|---|---|---|---|
| North | males | 2.0 | 2.1 | 2.5 | 2.2 |
| | females | 0.7 | 1.0 | 1.4 | 1.0 |
| | | (York) | (Wakefield) | (Preston) | |
| Central | males | 2.2 | 1.5 | 1.9 | 1.9 |
| | females | 1.0 | 0.9 | 1.3 | 1.1 |
| | | (Chester) | (Derby) | (Stoke) | |
| South | males | 2.8 | 1.0 | 2.0 | 1.9 |
| | females | 0.8 | 0.9 | 1.1 | 0.9 |
| | | (Ipswich) | (Plymouth) | (Newport) | |
| All latitudes: | | | | | |
| | males | 2.3 | 1.5 | 2.1 | |
| | females | 0.8 | 0.9 | 1.3 | |

women in the three better towns and lower in the three worse towns. The ratio of male to female incidence changed from 1.6 in the better towns combined to 1.3 in the intermediate towns to 0.8 in the worse towns. The decline in male incidence from "better" to "worse" towns may be summarised as a linear trend, which is statistically significant ($\chi^2 = 4.42$, 1 df, $P < 0.05$). The variation of female incidence with socio-economic status (highest average rates being in the "worse" towns) does not quite reach statistical significance at the 5% level ($\chi^2 = 5.63$, 2 df).

For carcinoma of the rectum (Table V) the male preponderance was present in every town. However the preponderance was greater in the better towns, where the sex ratio of incidences in the combined populations was 2.9, than in the intermediate and worse towns, where the sex ratios were 1.7. The variation of male incidence with socio-economic status (highest average rates being in the "better" towns) is statistically significant ($\chi^2 = 10.27$, 2 df, $P < 0.01$). The increase in female incidence from "better" to "worse" towns may be summarised as a linear trend, which is statistically significant ($\chi^2 = 5.00$, 1 df, $P < 0.05$).

Analysis by age group, 45–64 and 65–74 years, showed that for both carcinoma of the colon and rectum the variations with socio-economic status were maintained within each age group for both sexes.

When the survey data were cross-checked against Cancer Registrations marked discrepancies were revealed. (Cases aged 75 years and over were included in this check, although they were excluded from the main survey). For one town 65 cases of colon cancer were registered for 1979–80. Of these 23 were either incorrectly diagnosed, wrongly assigned to the county borough, or inadequately documented. Eight of the remaining 42 were not recorded in the survey: 2 had been diagnosed outside the town; the histology records of one were missing; 5 had not had histological confirmation of the diagnosis – 3 of them being older than 74 years. Eight cases recorded in the survey were not known to the cancer registry. For the other town the cancer registry provided 47 cases which met the diagnostic criteria of the survey. Five of them were not recorded in the survey. For all of these 5 the diagnosis was unconfirmed histologically: 4 were over the age of 74 years. Six of the 48 cases recorded in the survey were not registered. The survey methodology was therefore mainly fallible to underascertainment of cases over the age limit for inclusion.

Mortality rates for colonic cancer in the nine towns combined were 1.4 per 10,000 among men and 1.3 among women, compared with incidence rates of 1.9 and 1.6. Mortality for rectal cancer was 1.2 per 10,000 among men and 0.7 among women compared with incidences of 2.0 and 1.0. There was remarkably little variation in rectal cancer mortality, the range being 0.9–1.3 among men and 0.6–0.7 among women. In the nine towns the correlation coefficients between mortality rates and incidence were 0.14 for colonic cancer and 0.40 for rectal cancer.

The different pattern of incidence among men and women, according to the socio-economic status of the towns, was not reflected in the mortality data. Table VI shows the sex ratios of incidence and

**Table VI** Male/female ratios of incidence and mortality for colonic and rectal cancer, by social and economic conditions.

| | | *Social and economic conditions* | | |
|---|---|---|---|---|
| *Site* | *Rate* | *Better* | *Intermediate* | *Worse* |
| Colon | Incidence | 1.5 (1.0–2.3) | 1.3 (0.9–1.9) | 0.8 (0.6–1.2) |
| | Mortality | 1.3 (1.1–1.6) | 0.9 (0.7–1.1) | 1.1 (0.9–1.3) |
| Rectum | Incidence | 2.9 (1.8–5.0) | 1.7 (1.1–2.6) | 1.7 (1.2–2.5) |
| | Mortality | 1.8 (1.4–2.3) | 1.7 (1.3–2.1) | 1.8 (1.5–2.3) |

Figures in brackets are 95% confidence limits.

mortality, with corresponding 95% confidence limits, in the combined populations of the three better towns, the intermediate and the worse towns. The statistically significant trend in the sex ratio of incidence of colonic cancer is not shown by mortality, for which the ratios are 1.3, 0.9, and 1.1 in the better, intermediate and worse towns respectively. The high sex ratio of 2.9 for rectal cancer in the better towns is likewise not shown by mortality, for which the ratio is 1.8 in the better towns compared with 1.7 and 1.8 in the other two socio-economic groups.

### Discussion

This study has shown variations in the incidence of colorectal cancer in nine towns in England and Wales. Cases were identified by searching pathology records and, where necessary, additional information was obtained from clinical records. The main advantage of this method over the alternatives, namely cancer registry or mortality data, is the quality of the information on histology and site of lesion. In particular colonic and rectal lesions were distinguished with much greater precision than death certificate data allow – an important consideration in view of the evidence that the aetiologies of colonic and rectal cancers differ (*Lancet*, 1981). The main fallibility of the method is failure to detect cases who died without either histological confirmation of the disease during life or an autopsy. This possible source of error was reduced by restricting the survey to people aged less than 75 years. A cross-check against cancer registry data in two towns revealed under ascertainment of 14 % in the town selected for checking because of difficulties with pathology records, and 3% in the other town. Errors in the cancer registry data, due to both over and under-ascertainment, were considerably greater.

The pattern of variation in incidence differed for men and women so that colonic cancer had a male preponderance in affluent towns and a female preponderance in poor towns (Table IV) while correspondingly the male preponderance of rectal cancer was greater in the affluent than in the poor towns (Table V). These findings accord with the recent hypothesis that there are differing aetiological influences causing colorectal cancer in the two sexes (MacMichael & Potter, 1983). The hypothesis was based mainly on the different age distributions of the disease in the two sexes, women having proportionately higher rates at younger ages, and the different anatomical distribution within the large bowel, women having a greater proportion of proximal lesions. It was further suggested that these sex differences are due to intrinsic influences. Findings in three case control studies, taken together with the raised mortality from colon cancer among nuns, and the association between time trends in mortality and time trends in parity, suggest that parity is protective against colorectal cancer (Zaridze, 1983). Physiological changes associated with reproduction could influence a woman's risk of colon cancer through an effect mediated by changing bile acid secretion.

The higher rates of colorectal cancer among women in less affluent towns do not seem explicable by a negative association of the disease with parity. Family size is larger in these towns; married women aged 45–59 having an average number of 2.0 children in the better and intermediate towns and 2.1 in the worse towns (OPCS, 1971).

The sex differences in geographical distribution are not reflected in mortality (Table VI). The mortality rates are calculated from eleven years data and are therefore less susceptible to yearly fluctuations than the incidence data collected over two years. Nevertheless, the variations in male incidence are statistically significant for both carcinoma of the colon and rectum (Tables IV and V) and the variation in female incidence of carcinoma of the rectum is likewise significant. The trends in the sex ratio of incidence of colonic cancer (Table VI) are substantial in relation to the confidence limits: those for rectal cancer are less so, depending on the high figure for the better towns. There seems no obvious bias, such as different criteria for allocating lesions to the colon or rectum, which would explain the difference between incidence and mortality data. Nor do the different time periods for the data, 1968–78 for mortality and 1979–80 for incidence, offer a ready explanation. It is possible that there are differences in death certification practices in the towns, and this is being investigated. It is concluded, however, that mortality data are, in this instance, insufficiently sensitive to reveal a trend in incidence of the disease.

The results of international studies have suggested that low intake of dietary fibre may be a dominant influence in the aetiology of colorectal cancer. The findings in a mortality study in Britain support this (Bingham *et al.*, 1979). Regional mortality rates from colorectal cancer in Britain during 1969–73 were compared with estimates of fibre intake derived from the National Food Survey. There was an inverse correlation between mortality from colonic, but not rectal, cancer and the consumption of non-potato vegetables and pentosic fibre.

However the present study has shown that mortality from colorectal cancer does not closely reflect incidence. The correlation between colon cancer mortality and incidence was only 0.14. Furthermore interpretation of regional correlations between disease frequency and diet are made difficult by regions being large and heterogeneous geographical units. The regional mortality study does not therefore provide strong evidence in support of the fibre hypothesis.

Further doubt is cast by the geographical distribution of appendicitis, in whose aetiology low intake of dietary fibre is also suspected (Burkitt, 1971). A detailed study of acute appendicitis in the nine towns, using clinical and pathology records, showed a pattern of incidence clearly different to that of colorectal cancer, with higher incidences in the three northern towns within each socio-economic group and for both sexes (Barker & Liggins, 1981). This weighs against the hypothesis of similar, dominant dietary influences in the aetiology of both diseases.

We are grateful to the pathologists in the nine towns, who allowed us to use their records and without whose help this survey would not have been possible. We also thank the cancer registry staff who provided data. Dr Clive Osmond kindly gave statistical advice.

### References

EDITORIAL (1981). Large bowel cancer after cholecystectomy. *Lancet*, **ii**, 562.

BARKER, D.J.P., GARDNER, M.J., POWER, C. & HUTT, M.S.R. (1979). Prevalence of gallstones at necropsy in nine British towns. *Br. Med. J.*, **2**, 1389.

BARKER, D.J.P. & LIGGINS, A. (1981). Acute appendicitis in nine British towns. *Br. Med. J.*, **283**, 1083.

BINGHAM, S., WILLIAMS, D.R.R., COLE, T.J. & JAMES, W.P.T. (1979). Dietary fibre and regional large bowel cancer mortality in Britain. *Br. J. Cancer*, **40**, 456.

BURKITT, D.P. (1971). The aetiology of appendicitis. *Br. J. Surg.*, **58**, 695.

ELLIS, H. (1983). *Clinical Anatomy: A Revision and Applied Anatomy for Clinical Students*. Oxford: Blackwell Sci. Publ.

H.M.S.O. (1980). Report of the Advisory Committee on *Cancer Registration*; series MB1, **6**, 17.

MACMICHAEL, A.J. & POTTER, J.D. (1983). Do intrinsic differences in lower alimentary tract physiology influence the sex specific risks of bowel cancer and other biliary and intestinal diseases? *Am. J. Epidemiol.*, **118**, 620.

OFFICE OF POPULATION CENSUSES AND SURVEYS. (1971). *Small Area Statistics*. HMSO, London.

WILLIAMS, P.L. & WARWICK, R. (1980). *Grays Anatomy*. Edinburgh: Churchill Livingstone.

ZARIDZE, D.G. (1983). Environmental aetiology for large bowel cancer. *J. Natl Cancer. Inst.*, **70**, 389.